# Alterations in brain network functional connectivity and topological properties in DRE patients

**DOI:** 10.3389/fneur.2023.1238421

**Published:** 2023-12-05

**Authors:** Yongqiang Ding, Kunlin Guo, Jialiang Li, Qiao Shan, Yongkun Guo, Mingming Chen, Yuehui Wu, Xinjun Wang

**Affiliations:** ^1^Department of Neurosurgery, The Fifth Affiliated Hospital of Zhengzhou University, Zhengzhou, China; ^2^Henan Key Laboratory of Brain Science and Brain–Computer Interface Technology, School of Electrical and Information Engineering, Zhengzhou University, Zhengzhou, China; ^3^Department of Neurosurgery, The First People Hospital of Shangqiu, Shangqiu, China

**Keywords:** drug-resistant epilepsy, functional connectivity, network properties, EEG, graph theory

## Abstract

**Objective:**

The study aimed to find the difference in functional network topology on interictal electroencephalographic (EEG) between patients with drug-resistant epilepsy (DRE) and healthy people.

**Methods:**

We retrospectively analyzed the medical records as well as EEG data of ten patients with DRE and recruited five sex-age-matched healthy controls (HC group). Each participant remained awake while undergoing video-electroencephalography (vEEG) monitoring. After excluding data that contained abnormal discharges, we screened EEG segments that were free of artifacts and put them together into 20-min segments. The screened data was bandpass filtered to different frequency bands (delta, theta, alpha, beta, and gamma). The weighted phase lag index (wPLI) and the network properties were calculated to evaluate changes in the topology of the functional network. Finally, the results were statistically analyzed, and the false discovery rate (FDR) was used to correct for differences after multiple comparisons.

**Results:**

In the full frequency band (0.5–45 Hz), the functional connectivity in the DRE group during the interictal period was significantly lower than that in the HC group (*p* < 0.05). Compared to the HC group, in the full frequency band, the DRE group exhibited significantly decreased clustering coefficient (CC), node degree (D), and global efficiency (GE), while the characteristic path length (CPL) significantly increased (*p* < 0.05). In the sub-frequency bands, the functional connectivity of the DRE group was significantly lower than that of the HC group in the delta band but higher in the alpha, beta, and gamma bands (*p* < 0.05). The statistical results of network properties revealed that in the delta band, the DRE group had significantly decreased values for D, CC, and GE, but in the alpha, beta, and gamma bands, these values were significantly increased (*p* < 0.05). Additionally, the CPL of the DRE group significantly increased in the delta and theta bands but significantly decreased in the alpha, beta, and gamma bands (*p* < 0.05).

**Conclusion:**

The topology structure of the functional network in DRE patients was significantly changed compared with healthy people, which was reflected in different frequency bands. It provided a theoretical basis for understanding the pathological network alterations of DRE.

## 1 Introduction

Epilepsy is a common chronic neurological disease with a prevalence of 0.5–1% (1). Approximately 30% of patients with epilepsy who cannot effectively control seizure are classified as drug-resistant epilepsy (DRE). According to the International League Against Epilepsy definition (ILAE), the definition of DRE is the “failure of adequate trials of two tolerated, appropriately chosen and used antiseizure medication (ASM) schedules (whether as monotherapies or in combination) to achieve sustained seizure freedom,” which could be either three times the prior interictal interval or 1 year, whichever is longer (2). At present, the treatment of DRE remains a difficult challenge for physicians, and the quality of life of patients is often affected by cognitive and emotional disorders (3).

For patients with DRE, finding potential therapeutic targets and intervention strategies can effectively control seizures and improve the quality of life of patients (4). In this context, functional network analysis provides a powerful research tool. According to the network hypothesis, epileptic seizures can cause brain plasticity changes, including axonal sprouting, synaptic reorganization, and gliosis, which lead to the formation of abnormal neural networks (5). Furthermore, constructing functional networks based on neuroimaging or electrophysiological data can further clarify the impact of these changes on the overall functional characteristics of the brain (6). KM used resting-state magnetic resonance data to analyze the functional network of patients with epilepsy. The results showed that compared with healthy people, the functional network topology of patients with epilepsy was chaotic. The information transmission efficiency of the overall network pattern was significantly reduced, and it turned to a more random state (7).

Graph theory analysis is a common and important network analysis method when it comes to functional network research (8). It provides a visualization method for representing functional networks. Both the functional network is represented as nodes and edges to clearly show the connection relationship and topology between nodes. In addition, graph theory analysis also provides a series of metrics to describe the topological characteristics of functional networks (9). For example, characteristic path length and clustering coefficient represent the information transfer efficiency and local integration ability of the network. In Boris’s study, the functional networks of temporal lobe epilepsy patients and healthy people were compared, and the graph theory index was used to measure this difference. The results showed that the characteristic path length and clustering coefficient of patients increased, and the functional network was restructured in patients with temporal lobe epilepsy (10). Therefore, graph theory analysis plays an important role in the study of epilepsy network, which can help to understand the pathological mechanism of epilepsy and identify key nodes and functional modules so as to provide a reasonable basis for the prediction and control of epilepsy (11).

In this study, functional networks were constructed by collecting EEG data from DRE patients and healthy people. The graph theory analysis was used to measure the topological structure differences and functional mode changes between DRE and HC. We hope that this study will provide a reasonable basis for further exploring the functional network characteristics of DRE and improving treatment methods.

## 2 Materials and methods

### 2.1 Participants

A total of 10 patients diagnosed with DRE were enrolled in this study. Their clinical information was obtained from the electronic medical record system of the Fifth Affiliated Hospital of Zhengzhou University. The inclusion criteria were as follows: (1) the age of the subjects ranged from 18 to 60 years; (2) the patients had undergone a complete long-duration video electroencephalogram (VEEG) test lasting for more than 1 h; (3) the seizure and treatment were fully documented in the medical records; and (4) patients having no history of invasive brain surgery or non-invasive nerve stimulation. Additionally, we included five healthy adults who were matched for age and sex as controls. The control group was excluded if the patients (1) had a previous history of psychological disorders or seizures, (2) had abnormal brain structures in the magnetic resonance imaging (MRI), and (3) had a history of prior head trauma or surgery. Approximately 1 h of EEG data were collected from all healthy subjects while they were awake. This study was approved by the Ethics Committee of the Fifth Affiliated Hospital of Zhengzhou University (KY2020027).

### 2.2 Methods of EEG data collection

All participants were advised to avoid taking antiseizure medication and sedative drugs within 24 h before EEG acquisition to avoid influencing the analysis results. Throughout the EEG data collection process, participants were instructed to remain awake and were isolated in a quiet room. EEG data were recorded using the international 10-20 electrode placement system with the Nicolet system. The resistance of each recording electrode did not exceed 5 KΩ, and the binaural mastoid process was utilized as the reference electrode. The EEG data were uniformly sampled at a frequency of 500 Hz. For the recorded EEG data, we selected data from 19 electrodes (Fp1, Fp2, F3, F4, F7, F8, Fz, T3, T4, T5, T6, C3, C4, Cz, P3, P4, Pz, O1, and O2) for analysis.

### 2.3 Data selection and preprocessing

Two experienced neurophysiologists selected appropriate data from collected electroencephalogram (EEG) data. The data selection process was based on EEG waveforms and patient video recordings, following the following criteria: (1) EEG waveforms were stable, without abnormal sharp waves or slow waves; (2) participant video recordings showed closed eyes but alertness while sitting in a chair; and (3) electromyography (EMG) signals were stable, without significant artifacts. A 120-s resting state EEG segment was selected from each participant’s data and assembled into a 20-min fragment. For healthy participants, the data selection criteria were as follows: (1) no abnormal waveforms or abnormal leads in the EEG; (2) stable muscle EMG signals without significant interference artifacts. Approximately 240 s of data were selected for each healthy subject, composing a 20-min segment. Finally, the selected EEG data were preprocessed using the EEGLAB toolbox, with bandpass filtering ranging from 0.5 Hz to 45 Hz. Average reference and independent component analysis (ICA) were used for denoising and artifact removal. Based on computational considerations and the sampling frequency of the data, we divided the data into 2-s epochs for brain network construction. To conduct in-depth statistical compa.risons of brain network properties across different frequency bands, the EEG signals were bandpass filtered to standard frequency bands for analysis: δ (0.5–4 Hz), θ (4–8 Hz), α (8–13 Hz), β (13–30 Hz), and γ (30–45 Hz).

### 2.4 Construction of functional network

The research study has shown that the weighted phase lag index (wPLI) is highly sensitive to reducing the volume conduction effect while describing the synchronization of the electroencephalogram (EEG) time series (12). Therefore, in this study, wPLI was employed to quantify the functional connectivity strength between nodes and a functional brain network was constructed using the wPLI values between pairs of nodes. The specific calculation formula for wPLI is as follows:

(1) $$\mathrm{wPLI}=\frac{\left|E\left\{\left|\xi \left\{Y\right\}\mathrm{sgn}\left(\xi \left\{Y\right\}\right)\right|\right\}\right|}{E\left(\left|\xi \left\{Y\right\}\right|\right)}$$

where *Y* refers to the cross-spectrum of the two-time series and *ξ* represents the virtual portion of the cross-spectrum. The wPLI values are usually between 0 and 1, with 0 indicating no synchronization between the two time series and 1 indicating complete synchronization.

### 2.5 Calculation of network properties

We utilized the Brain Connectivity Toolbox (BCT) to compute the graph theory metrics of each network (13). Graph theory metrics are employed to assess the brain network’s small-worldness, i.e., whether it exhibits a high degree of clustering and short characteristic path lengths, features that enhance the efficiency of information transmission within the network. By calculating these metrics, we gain insights into the topological structure and information transfer characteristics of functional brain networks, aiding a deeper understanding of the brain’s functional connectivity patterns.

The primary metrics computed include the average node degree, average clustering coefficient, characteristic path length, and global efficiency of the network. The average degree (D) of the network is defined as follows:

(2) $$D=\frac{1}{N}\overset{N}{\underset{i=1}{\sum }}\overset{N}{\underset{j=1}{\sum }}w_{ij}$$

where N represents the number of nodes in the network and $w_{ij}$ represents the strength of the connection between node i and node j .

The clustering coefficient (CC) is typically used to measure the local connectivity and clustering characteristics of a network. It is defined by calculating the ratio between the actual number of connecting edges that exist between neighboring nodes near a given node i and the maximum possible number of connecting edges between neighboring nodes. The average clustering coefficient of the network is the mean of the clustering coefficients for N nodes, reflecting the degree of closeness in connections among all nodes in the network. The calculation formula is as follows:

(3) $$C=\frac{1}{N}\overset{N}{\underset{i=1}{\sum }}{\frac{2e_{i}}{m_{i}\left(m_{i}-1\right)}}_{i}$$

where $m_{i}$ is the number of nodes adjacent to node i , $e_{i}$ is the number of actual connection edges between nodes $m_{i}\text{,}$ and $m_{i}\left(m_{i}-1\right)/2$ is the maximum number of possible connection edges.

The characteristic path length (CPL) is the average of the shortest path lengths between all pairs of nodes in a network and is used to describe the network’s global properties. The formula for calculating the characteristic path length is as follows:

(4) $$CPL=\frac{1}{N\left(N-1\right)}\underset{i\neq j}{\sum }l_{ij}$$

where N is the number of all nodes in the network and $l_{ij}$ is the shortest path length between node i and node j .

Global efficiency (GE) is a global network characteristic used to measure the efficiency of information transfer within a network. The formula for calculating global efficiency is as follows:

(5) $$GE=\frac{1}{N\left(N-1\right)}\underset{i\neq j}{\sum }\frac{1}{l_{ij}}$$

### 2.6 Statistical analysis

Statistical tests were employed to identify significant differences in brain network connections and the graph theory metrics between the drug-resistant epilepsy (DRE) group and the healthy control (HC) group. First, an independent two-sample *t*-test was utilized to compare significant alterations in functional connectivity between brain regions in the DRE group relative to the HC group. Specifically, each connection between corresponding nodes in each network was represented by the weighted values of wPLI, resulting in a total of 600 weighted values across all networks. We conducted statistical tests on these 600 wPLI values between the two groups to discover significantly different brain region connections. To account for multiple comparisons between different brain regions in the wPLI matrix, false discovery rate (FDR) correction was applied (14). For the analysis of network properties, non-parametric rank-sum tests were employed for statistical assessment, with a significance level set at 0.05.

## 3 Results

### 3.1 Demographics and clinical data

The clinical information of ten DRE patients was collected, including gender, age, medical history, seizure type, and type of antiseizure medication (Table 1). All patients with DRE were prohibited from taking antiseizure medication for 24 h before receiving EEG data acquisition to avoid their effects on EEG activity (15).

**Table 1:** Clinical information of DRE patients.

Patients	Sex	Age	History	Type	Medicine
P1	Male	43	3	GTCS	VA + LMT
P2	Female	27	7	MAS	CBZ + LMT
P3	Male	20	4	GTCS	VA + TOP
P4	Female	30	10	CPS	VA + LCM
P5	Male	51	5	MAS	TOP+LCM
P6	Female	34	9	GTCS	VA + LMT
P7	Male	36	6	SPS	LMT + OXC
P8	Female	29	5	SPS	LMT + OXC
P9	Female	40	8	CPS	CBZ + LMT
P10	Female	39	12	GTCS	VA + TOP

VA, valproic acid; TOP, topiramate; CBZ, carbamazepine; LMT, lamotrigine; OXC, oxazepine; LCM, lacosamide; GTCS, generalized tonic–clonic seizures; MAS, myoclonic-atonic seizures; SPS, simple partial seizure; CPS, complex partial seizure.

### 3.2 Functional connectivity changes in the full-frequency band as well as in the sub-frequency band

**Figure 1:**
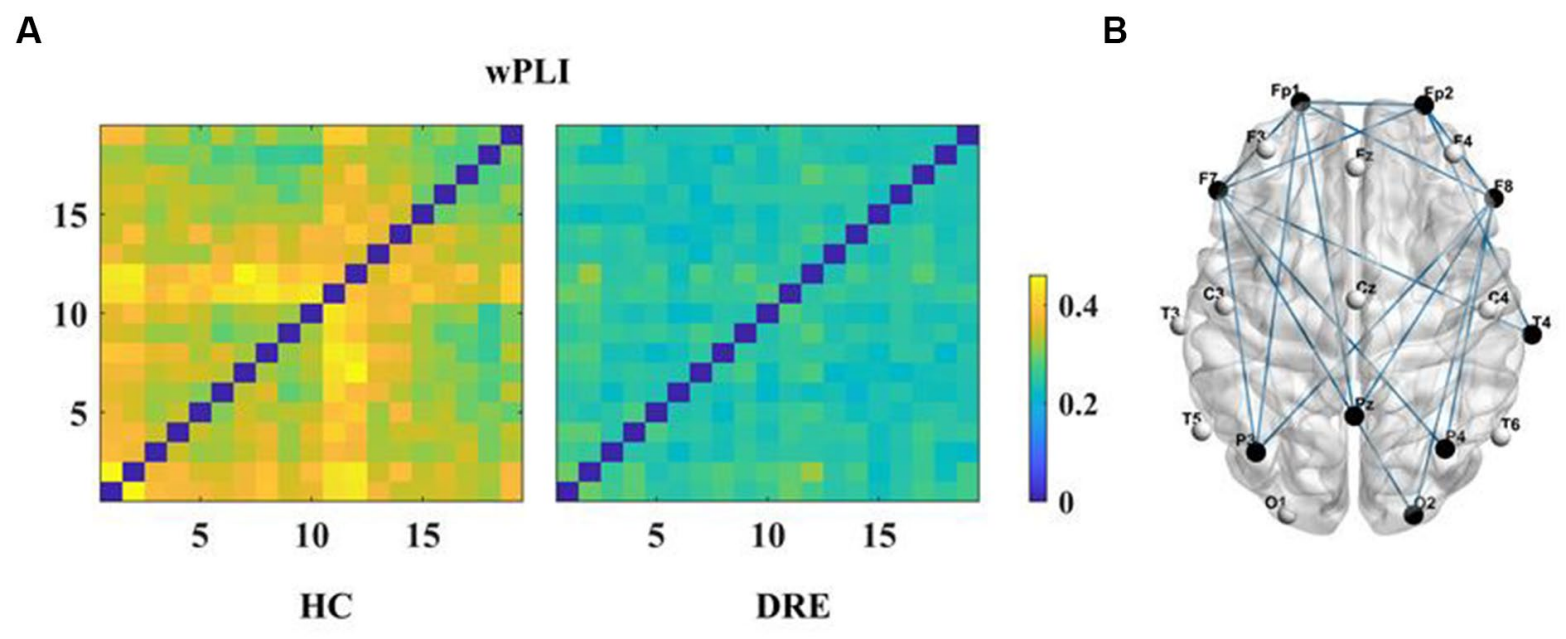
Functional connectivity and differences in the full frequency band. **(A)** Functional connectivity matrix for the full frequency band. **(B)** Significant differential connections in the DRE group compared to the HC group, where blue indicates connections significantly decreased in the DRE group relative to the HC group after multiple comparison correction (*p* < 0.05).

First, we constructed the functional connectivity matrix for the full-frequency band (0.5–45 Hz) (see Figure 1A) and conducted a statistical analysis of inter-nodal functional connections between the DRE group and the HC group to identify connections with significant differences. The results revealed a significant decrease in functional connectivity in the DRE group compared to the HC group across the full frequency band (*p* < 0.05). We presented the statistically significant differential connections in the top 10% of the results (see Figure 1B). The choice of using a 10% threshold was based on the fact that, after applying FDR correction, the remaining significantly different connections were fewer than 10% or 15%. This threshold selection ensured the retention of the majority of connections while excluding weaker ones. Furthermore, these significantly decreased connections were primarily located within the internal regions of the frontal lobe and between the frontal and parietal lobes. Simultaneously, we conducted the same analysis for non-drug-resistant epilepsy patients and drug-resistant epilepsy patients (see Supplementary Figures 1–5).

**Figure 2:**
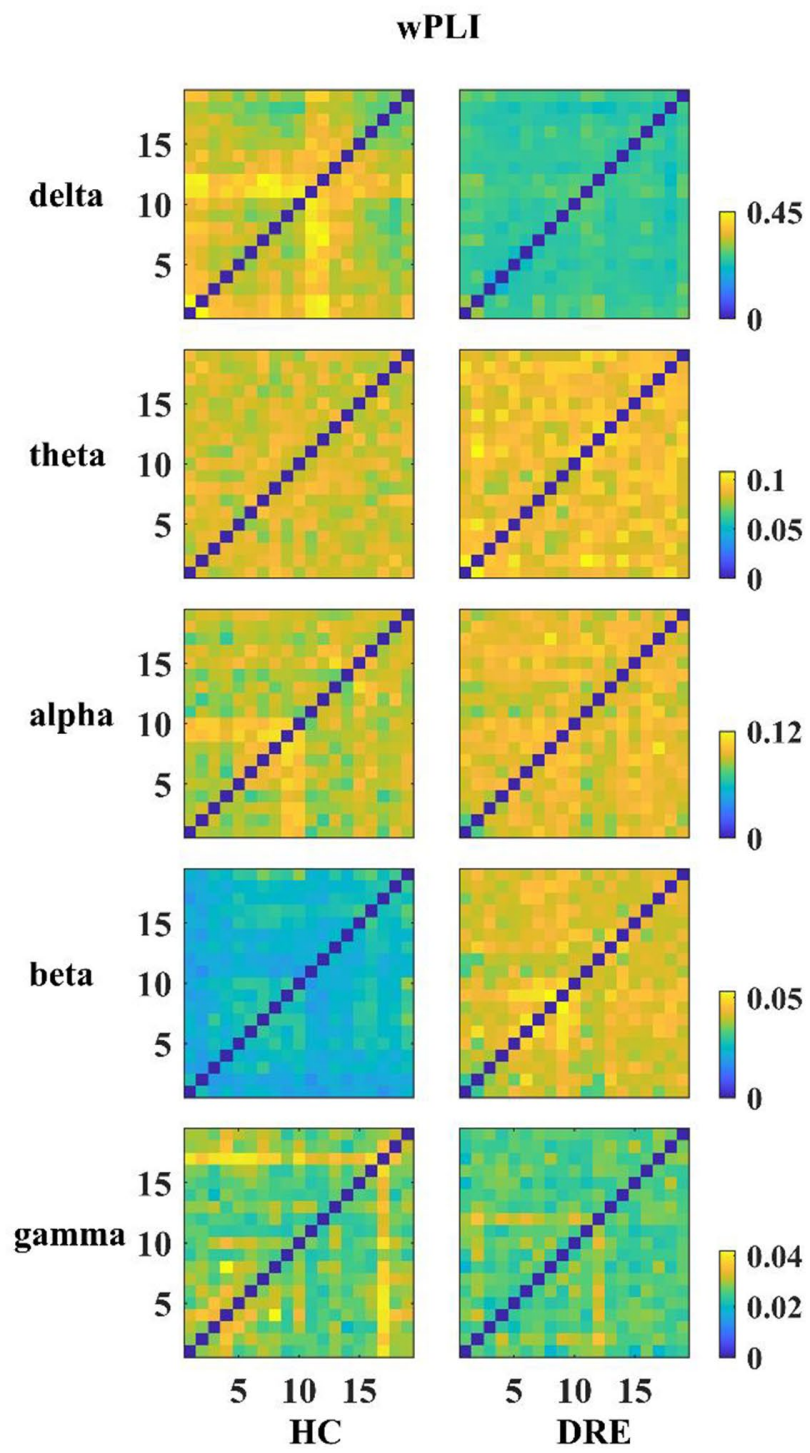
Functional connection matrix for each group in the different frequency bands.

Subsequently, to investigate differences in network connectivity within sub-frequency bands, we constructed functional connectivity matrices for DRE patients compared to the HC group using the same method within sub-frequency bands (see Figure 2).

**Figure 3:**
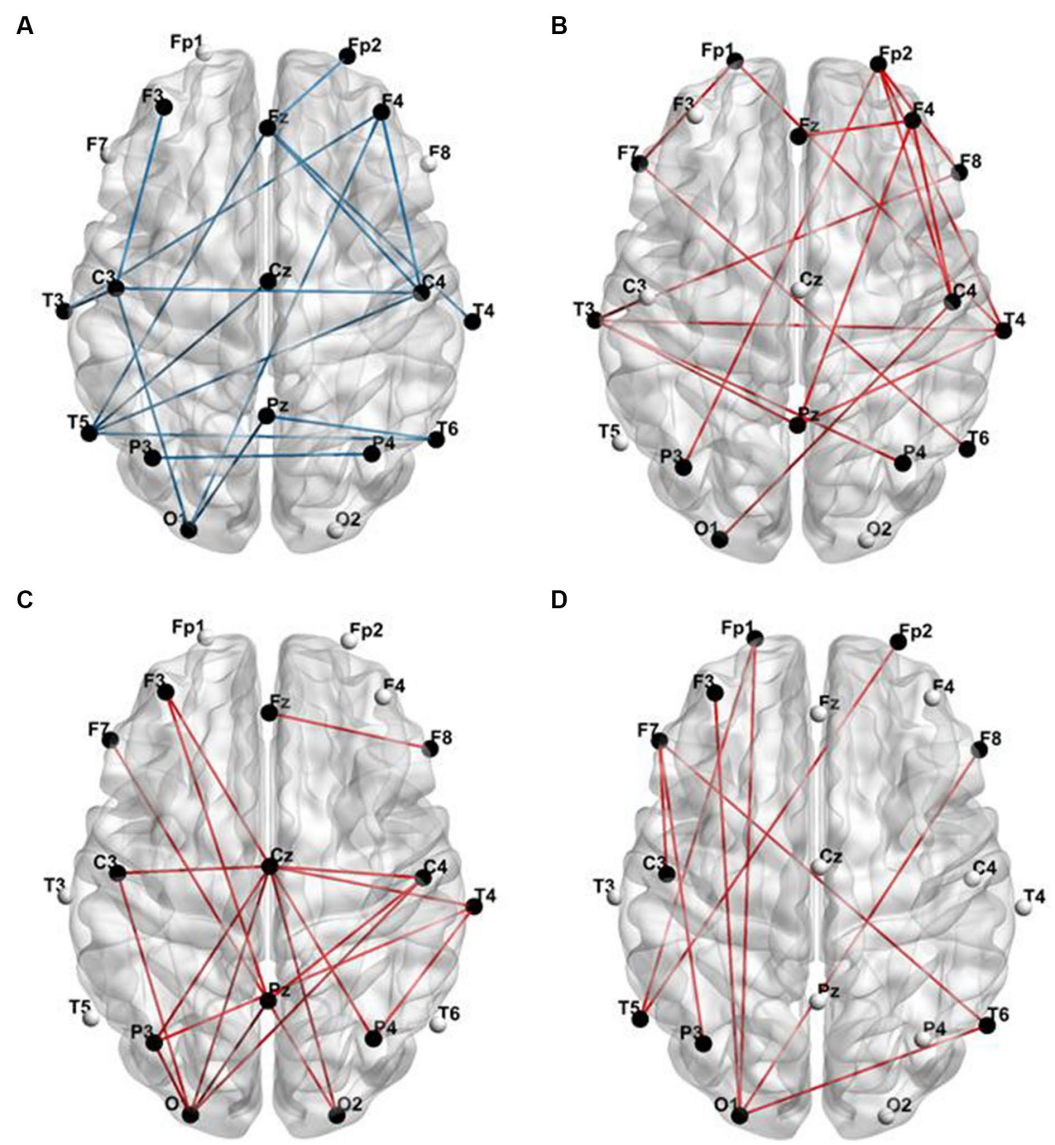
Functional connections with significant differences between the DRE group and the HC group. **(A)** Delta band; **(B)** alpha band; **(C)** beta band; **(D)** gamma band (red connections represent significantly enhanced functional connections, while blue connections represent significantly decreased functional connections at a *p-*value of <0.05).

In Figure 2, it is visually evident that the functional connectivity changes within sub-frequency bands for the DRE group relative to the HC group are not consistent. Functional connectivity decreases in the delta band, while it increases in the alpha, beta, and gamma bands. To quantitatively illustrate these differences, we have presented the statistically analyzed results (see Figure 3). In the figure, red connections represent significantly enhanced connections in the DRE group relative to the HC group, while blue connections indicate significantly decreased connections in the DRE group compared to the HC group. The results across frequency bands indicate that the changes in functional connectivity for the DRE group relative to the HC group are frequency dependent, with a significant decrease in the delta band (*p* < 0.05), no significant difference in the theta band (*p* > 0.05), and significant increases in the alpha, beta, and gamma bands (*p* < 0.05).

### 3.3 The network property changes in the full-frequency band and sub-frequency band

In order to further clarify the changes in the key features of the functional network of DRE patients. We calculated the network properties of the functional networks of the two groups and performed statistical analysis.

**Figure 4:**
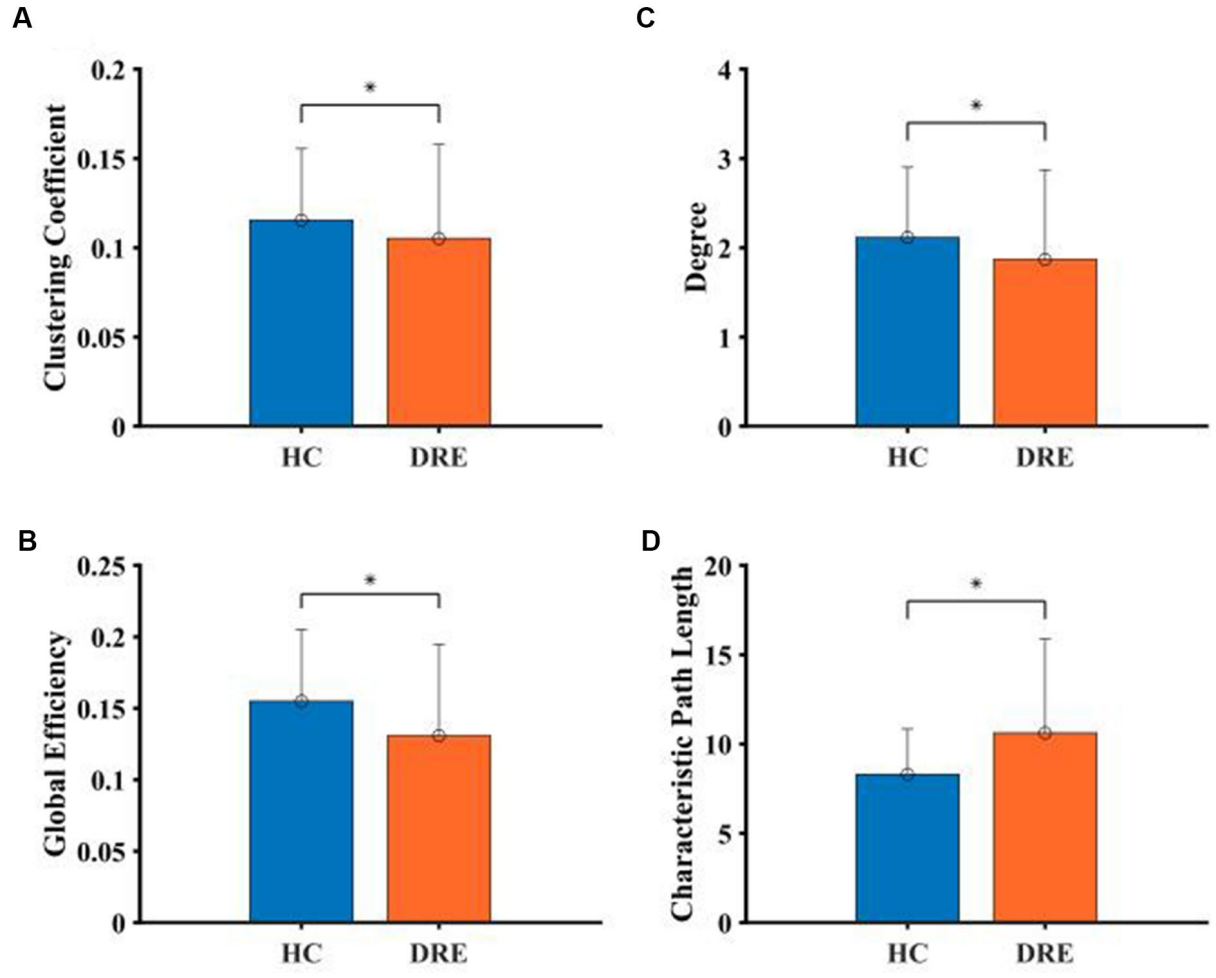
Comparison of network properties between the two groups in the full frequency band. **(A)** Clustering coefficient. **(B)** Degree. **(C)** Global efficient. **(D)** Characteristic path length (*indicated statistically significant difference at a *p*-value of <0.05).

The results of the full-band analysis showed that the functional network structure of DRE patients is more deviated from the small-world property than that of healthy people. Compared with healthy participants, CC, D, and GE in DRE patients were significantly decreased, while CPL was significantly increased (*p* < 0.05) (see Figure 4).

**Figure 5:**
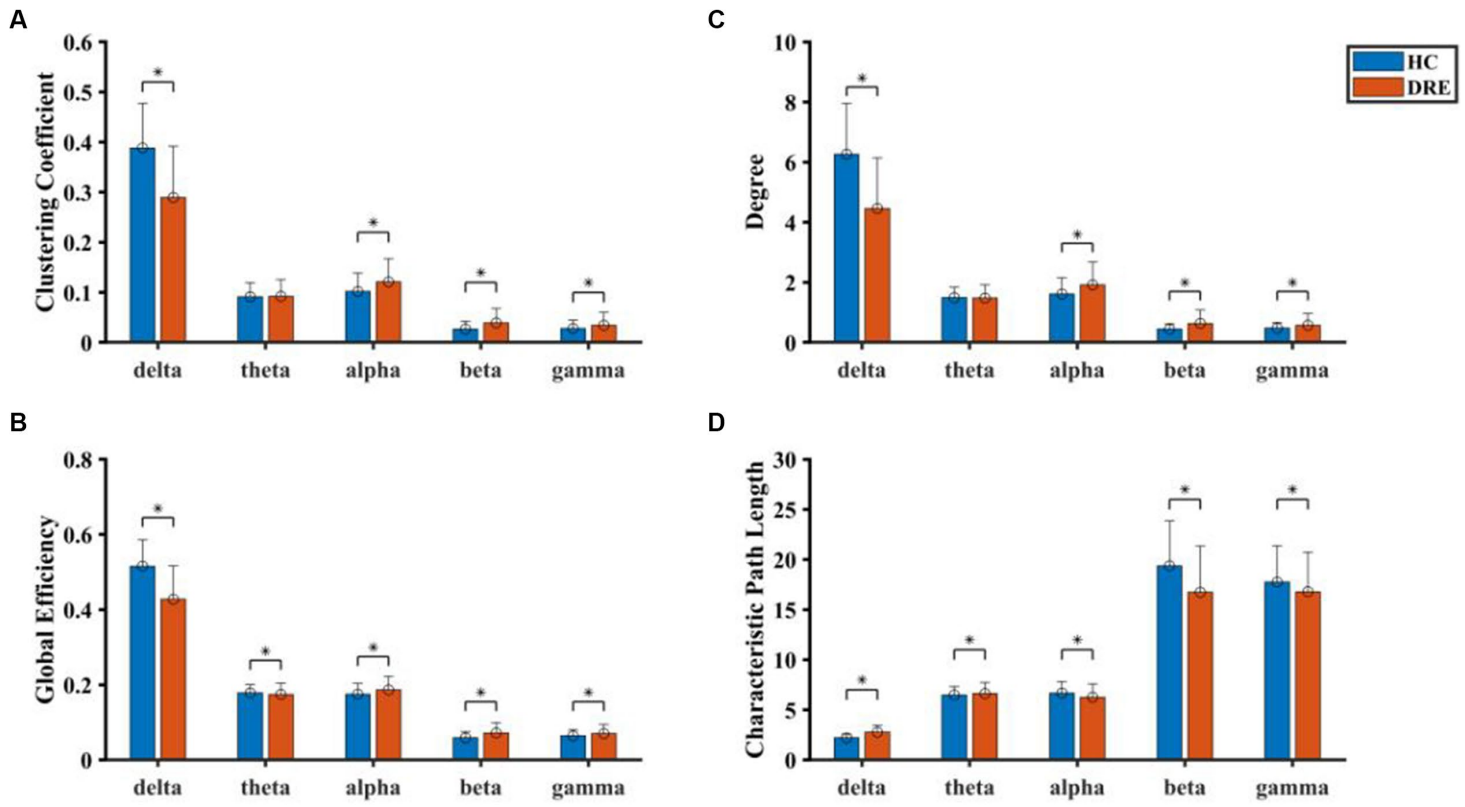
Comparison of network properties between the two groups in the sub-frequency band. **(A)** Clustering coefficient. **(B)** Degree. **(C)** Global efficient. **(D)** Characteristic path length (*indicated statistically significant difference at a *p*-value of <0.05).

In the sub-frequency band, the network properties were also different between the two groups of subjects. In the delta band, D, CC, and GE of DRE patients were significantly decreased compared with healthy participants, while CPL was significantly increased (*p* < 0.05). In the theta band, GE was significantly increased, and CPL was significantly decreased in DRE patients compared with healthy participants (*p* < 0.05). In alpha, beta, and gamma bands, DRE patients significantly increased D, CC, and GE, while CPL significantly decreased (*p* < 0.05) compared with healthy participants (see Figure 5).

## 4 Discussion

With the continuous development of the network hypothesis, people gradually realize that DRE is a disease involving complex brain network disorders (16). In this study, we collected interictal EEG data of DRE patients and healthy people to construct functional networks and calculated the network properties, including CC, D, GE, and CPL. However, after excluding spike–wave discharges, it was difficult to detect differences between EEG waveforms of DRE patients and healthy people by visual inspection (17). We found that the brain network of DRE patients was slightly decreased in functional connectivity and deviated from the small-world property compared with healthy people in the full band during the interictal period. This phenomenon provided an important basis for studying the pathological mechanism and treatment of DRE (18).

The change of synchrony in an epileptic patient brain network is a complex spatial dynamic process. At present, there is still controversy about the synchronization changes of functional network in epilepsy patients, which is mainly caused by different calculation methods and the types of experimental data (19). In Fernando’s study, a non-linear model was established to study the changes in synchrony in patients with epilepsy. The results showed that the degree of synchronization in the alpha band and beta band was significantly increased in patients with epilepsy (20). However, by collecting EEG data of patients with temporal lobe epilepsy and healthy people and constructing functional networks for comparison. Liao et al. found that the degree of synchronization in the alpha, beta, and gamma bands of patients with temporal lobe epilepsy was significantly decreased (21).

In this study, it was found that the synchronization of DRE patients in the full frequency band was reduced compared with healthy people. However, in different frequency bands, the changes in patient synchrony showed the opposite trend. The synchrony decreased in the delta band and increased in the alpha, beta, and gamma bands. In the neural network of DRE patients, the synaptic remodeling between neurons leads to abnormal structural and functional connectivity (12). This affects the transmission of information and reduces the degree of synchronization. In addition, due to the lack of effective control of seizures, the patient’s neuronal activity will appear as abnormal synchronicity and rhythmicity (13). Previous studies have found that different frequency bands are related to the symptoms of patients with epilepsy (14). The synchronization changes in the delta band reflect the disturbance of consciousness and cognitive impairment of patients, while the synchronization changes in the beta and gamma bands reflect the abnormal synchronization and severity of seizures (22). Therefore, the results of the present study may suggest a relationship between the clinical manifestations and the degree of network synchronization in DRE patients, with the decrease of delta band synchronization associated with the impairment of consciousness and cognitive function, and the increase of beta and gamma band synchronization may be associated with recurrent seizures.

According to the results of the study, the CC, D, and GE of DRE patients decreased while CPL increased in the full band. This trend indicated that the local aggregation and interconnection of nodes in the functional network of DRE patients decreased (23). At the same time, the increase in the average path length between nodes reduces the efficiency of information transmission in the global scope. In previous studies, by comparing the functional network structure of patients with temporal lobe epilepsy and healthy people, it was found that the increase in CC and CPL in patients with epilepsy led to the development of a functional network with a more regular structure (24). This regular network structure was more vulnerable to the influence of abnormal discharge compared with the small-world network (10). However, the network properties of DRE patients showed different changes in our study, which indicated that the network structure of DRE patients did not change to the regular network but only deviated from the small-world property.

In the comparison of network properties in different frequency bands, the functional network structure on the delta band deviated from the small-world network. However, the functional network structure of the alpha band, beta band, and gamma band was more inclined to a small-world network. This phenomenon complemented the changes in functional connectivity in the network. Compared with functional connectivity, the network properties can further evaluate the stability of the overall structure of the brain network (25). Summarizing the above results, we found that the network properties of DRE patients were further enhanced based on the increased functional connectivity. This result was similar to previous studies in that the increased functional connectivity between brain regions represented the increased coupling of neuronal activities, and the network structure was biased toward the mode transition with higher information transmission efficiency (26).

Considering that the study of functional networks in patients with epilepsy is easily interfered by related factors, we need to further discuss the experimental procedures and the application of computational methods in this study to make the results representative (27). First, considering the influence of ASM on EEG results, ASM and sedative drugs were prohibited for all participants within 24 h before data collection to avoid their influence on the experimental results (28). Although we have tried our best to avoid the interference of drugs, the blood concentration of ASM in participants may still exceed the normal value. Therefore, we will further discuss the influence of ASM on the results of functional network analysis in the follow-up study (29). Since a 19-channel lead system was used for the acquisition of EEG data in this study, the calculated network properties include CC, D, CPL, and GE. However, the small world index (SWI) was not used to measure the topological changes of functional networks (30). This is mainly due to the fact that EEG data containing only 19-channel leads cannot provide sufficient spatial information about the network, thus limiting the accurate assessment of the network topology (31). In addition to this, using only 19-channel lead EEG data to calculate SWI may miss some potentially important brain region connections. Because these connections may be located in regions beyond the range of the selected channel, this may lead to a lack of confidence in the results of the calculation of the small-world indicator (32).

In this study, wPLI values and network properties were computed by segmenting the data into appropriate time lengths. However, it is worth noting that different computational methods and epoch lengths can have an impact on the construction of functional networks (33). Moreover, the temporal characteristics of frequency bands in different frequency ranges vary, which may result in the final analysis results not fully capturing the periodic characteristics of EEG (34). Therefore, we compared the functional connectivity of the HC group in the delta band at a 500 Hz sampling rate, including epochs of 2 s, 4 s, and 8 s, as well as at a 256 Hz sampling rate with 4-s epochs and a 128 Hz sampling rate with 8-s epochs. The results showed that at a sampling rate of 500 Hz, the wPLI values gradually decreased with increasing epoch length (see Supplementary Figure 6). When the epoch length was the same, there were no significant differences in functional connectivity, even with different sampling rates (see Supplementary Figure 7). Although functional connectivity significantly decreased with increasing epoch length, the synchrony trend in the network did not change significantly. Bai et al. used scalp EEG data to compare the interictal functional connectivity of temporal lobe epilepsy patients at different time scales and found that both showed a similar trend of changes (35). Therefore, considering sampling rates and computational load, we opted for 2-s EEG segments for our analysis to strike a balance between temporal resolution and estimation stability, while ensuring an adequate sample size (33). The 2-s time window also provided better coverage of functional connectivity changes across various time scales.

In this study, by comparing the functional network of patients with drug-resistant epilepsy (DRE) and healthy individuals, it was demonstrated that there are significant differences in the functional network of DRE patients relative to healthy individuals. The results showed that the interictal functional network in DRE patients was synchronously decreased, and the network structure deviated from the small-world property. However, due to the small sample size included in this study and the relatively simple calculation method adopted, the spatial features of EEG were not further explored. Therefore, a large number of neuroimaging and electrophysiological data of DRE patients need to be further included in future studies to help explore the pathological mechanism and treatment options of DRE.

## Data availability statement

The original contributions presented in the study are included in the article/Supplementary material, further inquiries can be directed to the corresponding authors.

## Ethics statement

The studies involving humans were approved by the Ethics Committee of the Fifth Affiliated Hospital of Zhengzhou University. The studies were conducted in accordance with the local legislation and institutional requirements. Written informed consent for participation was not required from the participants or the participants’ legal guardians/next of kin in accordance with the national legislation and institutional requirements.

## Author contributions

YD: study concept and design, and acquisition of data. KG: data analysis, interpretation, and drafting. YG and QS: study coordination. JL: study design, interpretation of data, and revising the article for content. MC, YW, and XW: study concept and design and revising the article for content. All authors contributed to the article and approved the submitted version.
