# Supplementary material for: Alterations in brain network functional connectivity and topological properties in DRE patients
Source: Front Neurol. 2023 Dec 5;14:1238421. doi: 10.3389/fneur.2023.1238421 (PMC10729765; doi:10.3389/fneur.2023.1238421)
Supplement: Supplementary file 1 [file Data_Sheet_1.PDF]

The supplementary materials aim to provide comparative analysis results of functional network connectivity and network properties between Drug-Sensitive Epilepsy (DSE) patients and drug-resistant epilepsy (DRE) patients. Additionally, it includes the analysis results of constructing functional networks with different epoch lengths and the same epoch length but different sampling frequencies.

Supplemental Information

Functional connectivity changes in the full frequency band as well as in the sub-frequency band

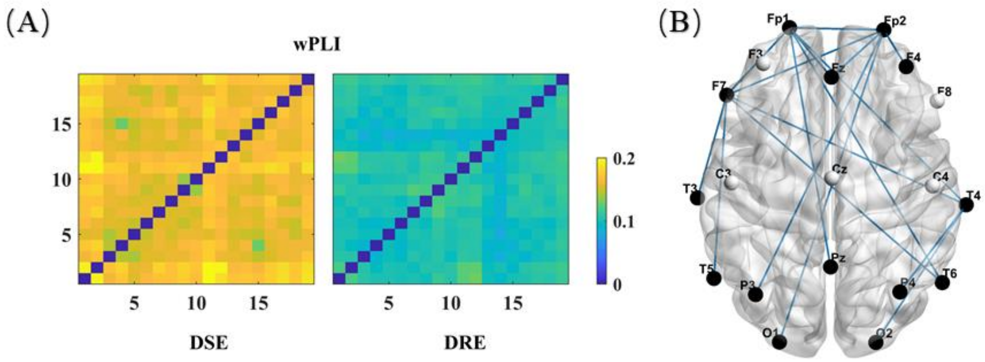

FIGURE 1 | Functional connectivity and differences in the full frequency band. (A): Functional connectivity matrix for the full frequency band; (B) Significant differential connections in the DRE group compared to the DSE group, where blue indicates connections significantly decreased in the DRE group relative to the DSE group after multiple comparison correction. $P < 0.05$.

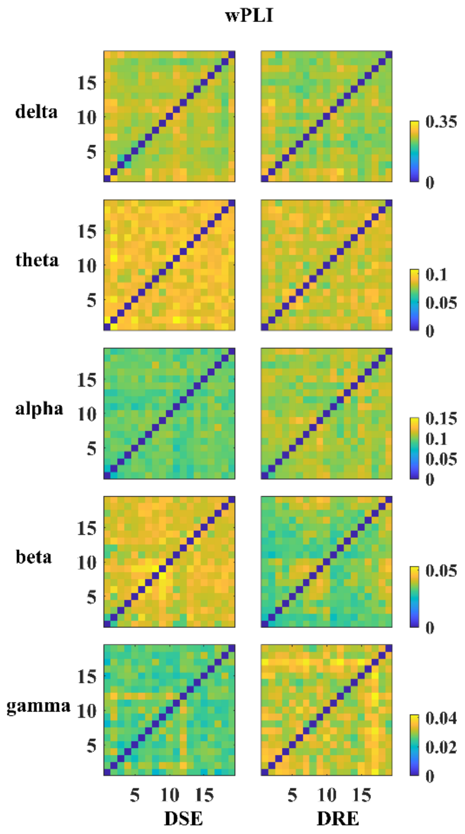

FIGURE 2 | The functional connection matrix for each group in the different frequency bands.

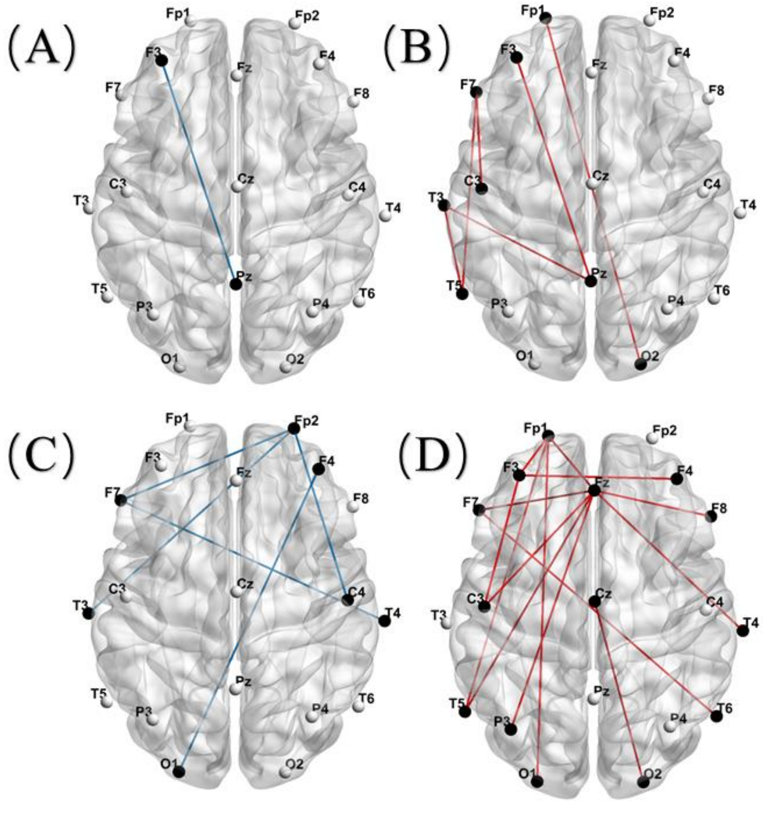

FIGURE 3 | Functional connections with significant differences between the DRE group

and the DSE group. (A) Delta band; (B) Alpha band; (C) Beta band; (D) Gamma band. (Red connections represent significantly enhanced functional connections, while blue connections represent significantly decreased functional connections) $P < 0.05$.

The network properties changes in the full frequency band and in the sub-frequency band

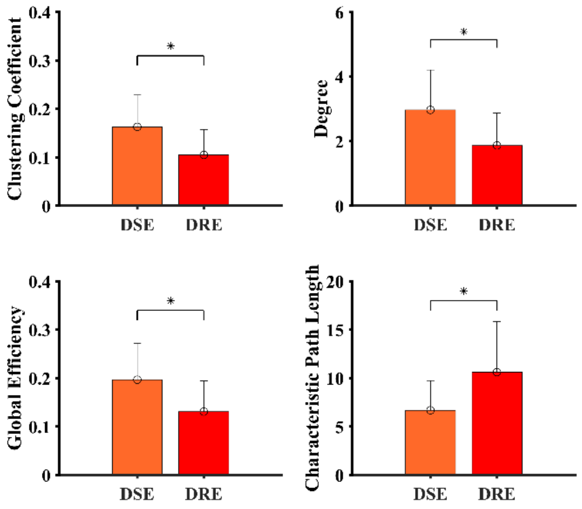

FIGURE 4 | Comparison of network properties between the two groups in the full frequency band (*indicated statistically significant difference $P < 0.05$).

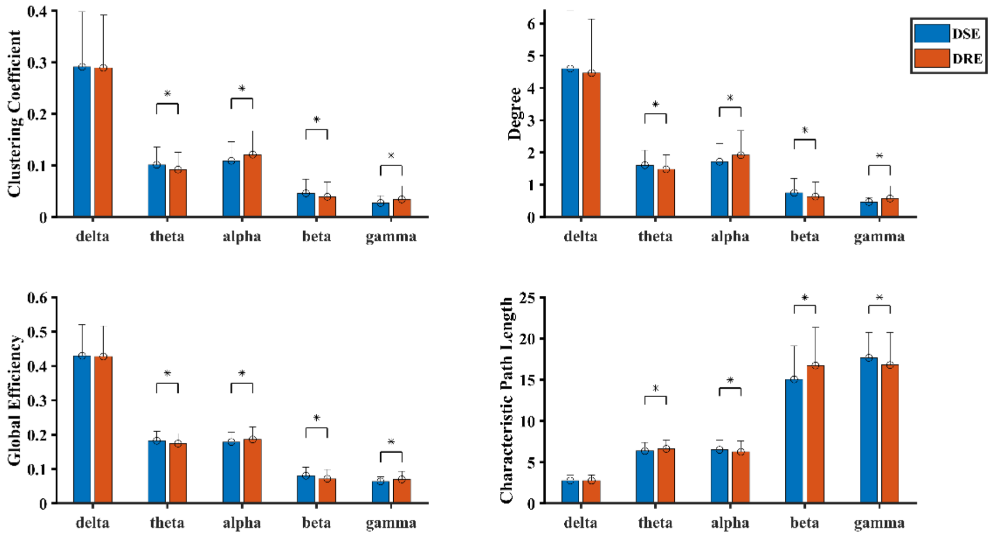

FIGURE 5 | Comparison of network properties between the two groups in the sub-frequency band (* indicated statistically significant difference $P < 0.05$).

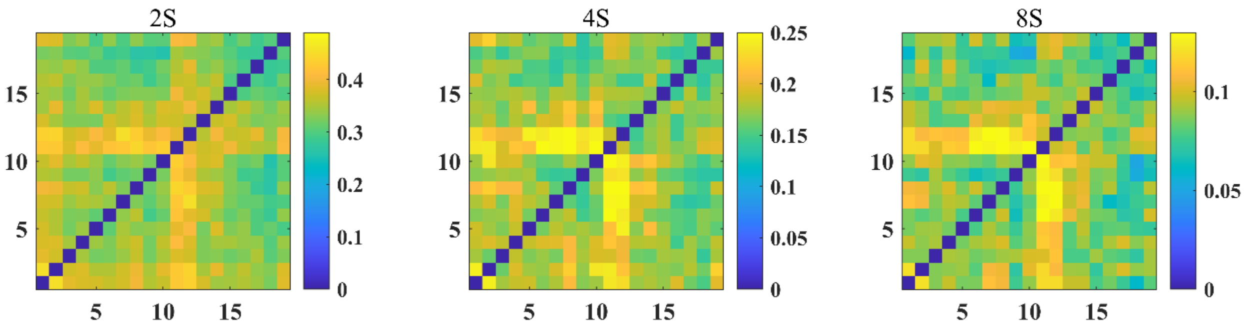

FIGURE 6 | Functional connectivity matrices with different epoch lengths in the delta band of the HC group at a sampling rate of 500Hz.

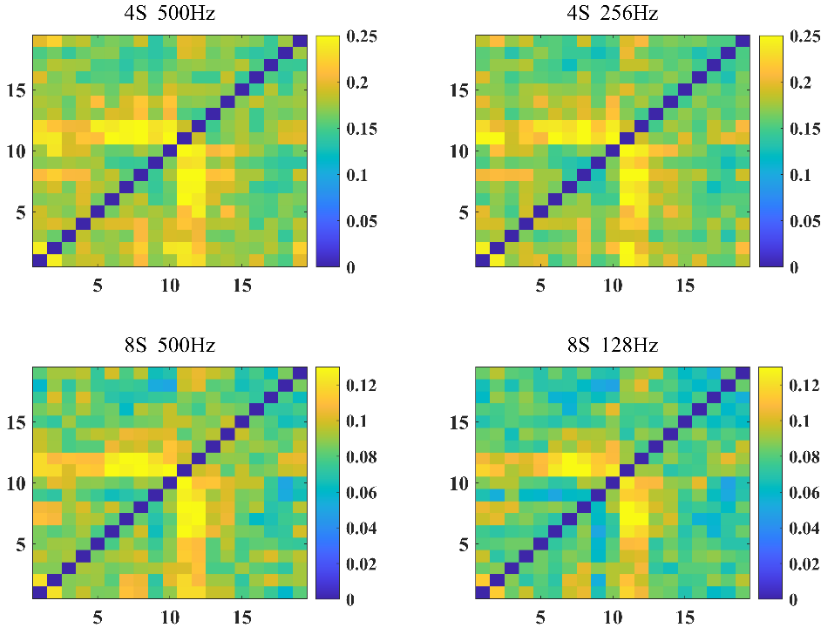

FIGURE 7 | Functional connectivity matrices with the same epoch length but different sampling frequencies.
